# Supplementary material for: Mutations in PCYT2 disrupt etherlipid biosynthesis and cause a complex hereditary spastic paraplegia
Source: Brain. 2019 Oct 22;142(11):3382–97. doi: 10.1093/brain/awz291 (PMC6821184; doi:10.1093/brain/awz291)
Supplement: awz291_Supplementary_Data [file awz291_supplementary_data.zip › awz291-Suppl_data/Supplementary_Data4.zip!/Supplementary information 4 - plasma lipidomics.zip!/Plasma lipidomics/Control vs Patient1/Statistical plots and data/Volcano plot.pdf]

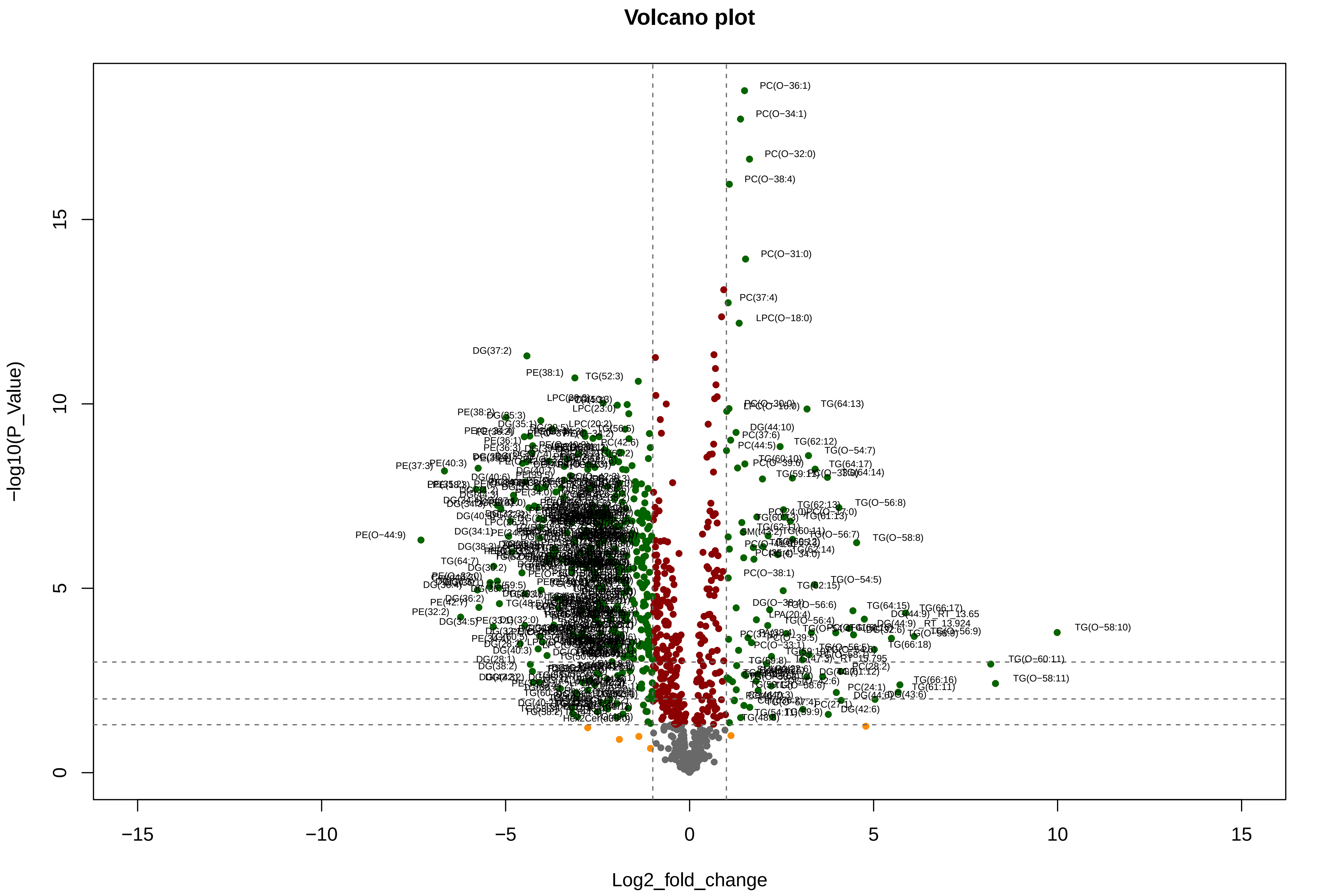

Volcano plot
−log10(P_Value)
Log2_fold_change
0
5
10
15
−15
−10
−5
0
5
10
15
PC(O−36:1)
PC(O−34:1)
PC(O−32:0)
PC(O−38:4)
PC(O−31:0)
PC(37:4)
LPC(O−18:0)
DG(37:2)
PE(38:1)
TG(52:3)
LPC(23:0)
LPC(20:2)
TG(56:5)
PC(42:6)
PE(38:2)
DG(35:3)
DG(35:1)
PE(36:1)
PE(36:3)
PE(37:3)
PE(40:3)
DG(40:6)
DG(40:7)
DG(34:1)
PE(O−44:9)
DG(38:3)
TG(64:7)
DG(30:2)
TG(59:5)
DG(36:2)
PE(42:7)
PE(32:2)
DG(34:5)
PE(33:1)
DG(32:0)
DG(40:3)
DG(28:1)
DG(38:2)
TG(58:2)
TG(64:13)
DG(44:10)
PC(37:6)
PC(44:5)
TG(62:12)
TG(O−54:7)
TG(64:17)
TG(64:14)
TG(59:11)
TG(62:13)
TG(O−56:8)
TG(61:13)
TG(O−56:7)
TG(O−58:8)
TG(62:14)
PC(O−34:0)
PC(O−38:1)
TG(O−54:5)
TG(62:15)
DG(O−38:4)
TG(O−56:6)
LPA(20:4)
TG(O−56:4)
TG(64:15)
TG(66:17)
TG(O−56:9)
TG(O−58:10)
TG(66:18)
PC(O−33:1)
TG(O−60:11)
PC(28:2)
TG(O−58:11)
TG(66:16)
TG(61:11)
PC(24:1)
DG(44:6)
DG(43:6)
DG(42:6)
PC(27:1)
TG(59:9)
